# Novel nikkomycin analogues generated by mutasynthesis in *Streptomyces ansochromogenes*

**DOI:** 10.1186/1475-2859-13-59

**Published:** 2014-04-21

**Authors:** Chi Feng, Hongbo Ling, Deyao Du, Jihui Zhang, Guoqing Niu, Huarong Tan

**Affiliations:** 1 State Key Laboratory of Microbial Resources, Institute of Microbiology, Chinese Academy of Sciences, NO.1 Beichen West Road, Chaoyang District, Beijing 100101, China; 2 University of Chinese Academy of Sciences, No.19A Yuquan Road, Beijing 100049, China; 3 Present address: Department of Molecular Oncology, H. Lee Moffitt Cancer Center and Research Institute, 12902 Magnolia Drive, Tampa, FL 33612, USA

**Keywords:** Novel analogues, Mutasynthesis, *sanL* mutant, *Streptomyces ansochromogenes*

## Abstract

**Background:**

Nikkomycins are competitive inhibitors of chitin synthase and inhibit the growth of filamentous fungi, insects, acarids and yeasts. The gene cluster responsible for biosynthesis of nikkomycins has been cloned and the biosynthetic pathway was elucidated at the genetic, enzymatic and regulatory levels.

**Results:**

*Streptomyces ansochromogenes* ΔsanL was constructed by homologous recombination and the mutant strain was fed with benzoic acid, 4-hydroxybenzoic acid, nicotinic acid and isonicotinic acid. Two novel nikkomycin analogues were produced when cultures were supplemented with nicotinic acid. These two compounds were identified as nikkomycin Px and Pz by electrospray ionization mass spectrometry (ESI-MS) and nuclear magnetic resonance (NMR). Bioassays against *Candida albicans* and *Alternaria longipes* showed that nikkomycin Px and Pz exhibited comparatively strong inhibitory activity as nikkomycin X and Z produced by *Streptomyces ansochromogenes* 7100 (wild-type strain). Moreover, nikkomycin Px and Pz were found to be more stable than nikkomycin X and Z at different pH and temperature conditions.

**Conclusions:**

Two novel nikkomycin analogues (nikkomycin Px and Pz) were generated by mutasynthesis with the *sanL* inactivated mutant of *Streptomyces ansochromogenes* 7100. Although antifungal activities of these two compounds are similar to those of nikkomycin X and Z, their stabilities are much better than nikkomycin X and Z under different pHs and temperatures.

## Introduction

Nikkomycins are a group of peptidyl nucleoside antibiotics produced by *Streptomyces ansochromogenes*[1] and *Streptomyces tendae*[2]. Acting as competitive inhibitors of chitin synthase, nikkomycins inhibit the growth of filamentous fungi, insects, acarids and yeasts [3]. The gene cluster responsible for biosynthesis of nikkomycins has been cloned from *S. ansochromogenes* and *S. tendae*. Biosynthesis of nikkomycins has been studied extensively at the genetic, enzymatic and regulatory levels in both *Streptomyces* strains [4-11].

**Figure 1:**
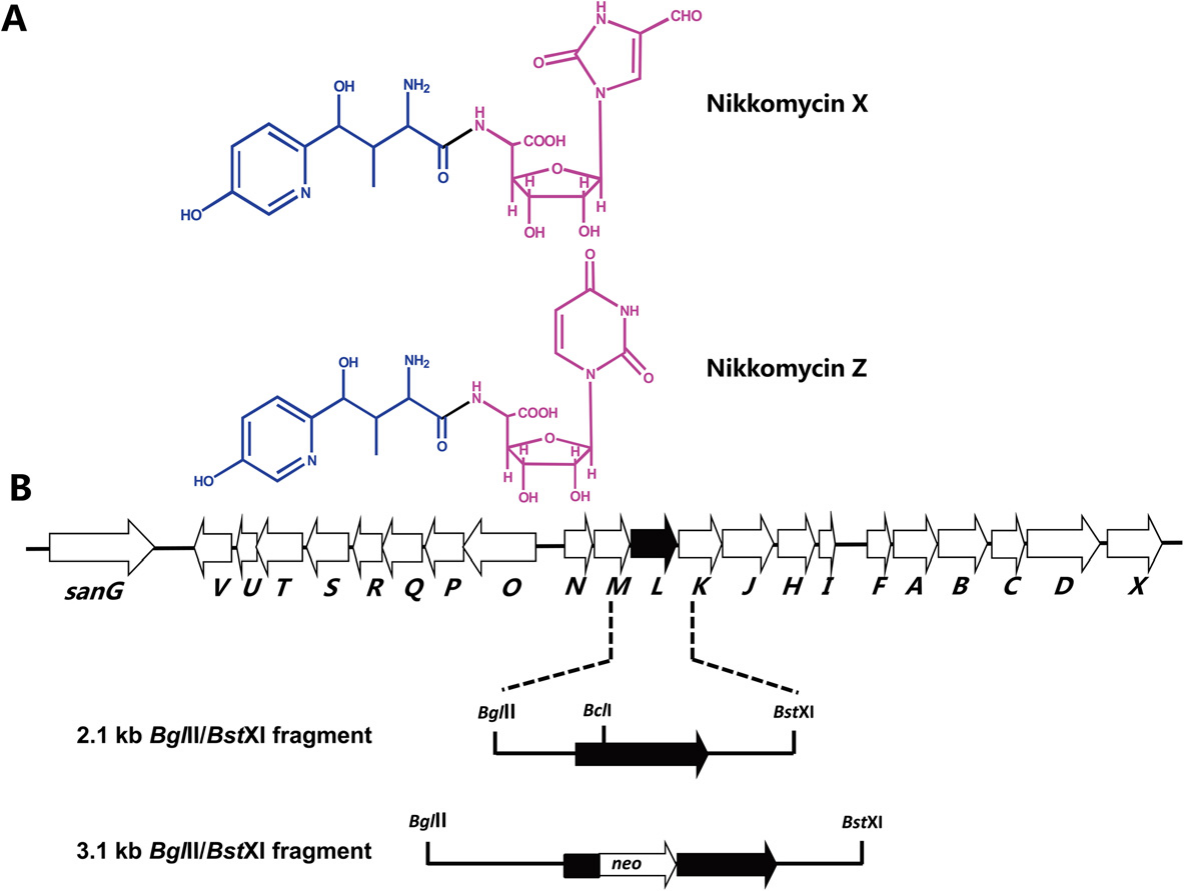
**Chemical structure (A) and organization of the gene cluster for nikkomycin biosynthesis (B).** The peptidyl moiety (HPHT) and nucleoside moiety of nikkomycin were indicated by blue color and red color, respectively. The solid arrow shows *sanL* and its orientation. The 2.1 kb *Bgl*II/*Bst*XI fragment contains *sanL* and its flanking sequences. The 3.1 kb *Bgl*II/*Bst*XI fragment was used for disruption of *sanL*. The kanamycin resistance gene (*neo*) was inserted into *Bcl*I site of *sanL*.

Nikkomycins are composed of peptidyl and nucleoside moieties. The peptidyl moiety consists of 4-(4′-hydroxy-2′-pyridinyl)-homothreonine (HPHT) while the nucleoside moiety varies in different nikkomycins (Figure 1A). Previous studies demonstrated that a L-lysine 2-aminotransferase catalyzes the initial reaction from L-lysine to piperideine-2-carboxylate (P2C) in HPHT formation [9]. A monomeric sarcosine oxidases was found to be responsible for the conversion of P2C to picolinic acid, which was activated by a picolinate-CoA ligase to continue subsequent reactions [4,10].

Mutasynthesis has become a useful method in the generation of new antibiotic derivatives. It can be started by blocking the biosynthesis of key biosynthetic intermediates, and a variety of alternative intermediates can then be fed to the mutant to produce novel antibiotic derivatives [12]. This approach could expand the chemical diversity of antibiotics and produce novel compounds with improved pharmaceutical properties [13,14].

Here we described the mutasynthesis of nikkomycins Px and Pz by feeding *S. ansochromogenes* ΔsanL with analogues of picolinic acid. Supplementation of the mutant strain with nicotinic acid led to production of two novel nikkomycin analogues which exhibited improved properties.

## Results

### Construction of *sanL* inactivation mutant and its complementation

**Figure 2:**
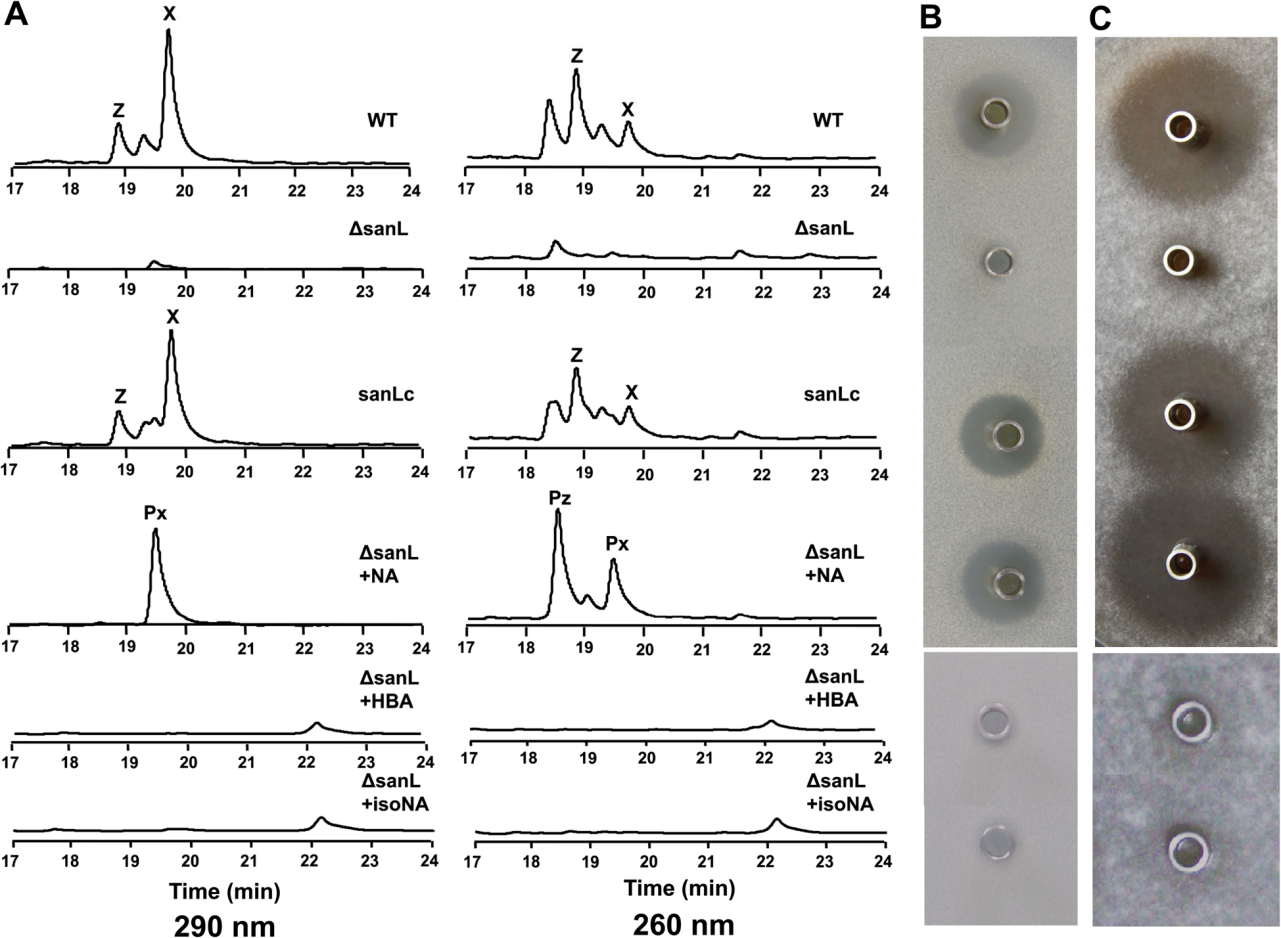
**HPLC analysis and bioassay of nikkomycin Px and Pz.** HPLC analysis of fermentation broth from *S. ansochromogenes* 7100 and its derivatives **(A)** and bioassays of nikkomycin production against *C. albicans ***(B)** and *A. longipes ***(C)**. WT: *S. ansochromogenes* 7100, ΔsanL: *sanL* disruption mutant, sanLc: *sanL* complementary strain, NA: nicotinic acid, HBA: 4-hydroxybenzoic acid, isoNA: isonicotinic acid, X: nikkomycin X, Z: nikkomycin Z, Px:nikkomycin Px, Pz: nikkomycin Pz.

Mutasynthesis requires the generation of mutants that are blocked in the formation of key biosynthetic intermediate of the end-product. For this purpose, we inactivated *sanL* by double-crossover recombination in *S. ansochromogenes* 7100 (Figure 1B). Sequence analysis revealed that *sanL* encodes a L-lysine 2-aminotransferase which showed 97% identity with NikC from *S. tendae*. The disruption of *sanL* was confirmed by PCR and Southern blot (data not shown). The resulting inactivated mutant was subjected to HPLC analysis to assess nikkomycin production. In comparison with nikkomycin production in the wild-type strain, no nikkomycin was detected in the fermentation medium of *sanL* mutant (ΔsanL) (Figure 2A). To test the antifungal activity of ΔsanL strain, the culture filtrates of ΔsanL and wild-type strains were subjected to bioassays against *C. albicans* and *A. longipes.* Culture filtrate of ΔsanL lost the ability to inhibit the growth of *C. albicans* and *A. longipes* (Figure 2B and C). Because *sanL* is situated within a group of 7 genes that form an operon, inactivation of downstream genes by polar effects on transcription might have contributed to the phenotype observed. To exclude this possibility, the ΔsanL strain was complemented by pSET152::*sanL* in which a single copy of *sanL* was driven by the constitutive *hrdB* promoter. The resulting complementation strain (sanLc) restored nikkomycin production (Figure 2A) and its antifungal activities (Figure 2B and C).

### Feeding of ΔsanL strain with analogues of picolinic acid

Previous studies suggested that the picolinate-CoA ligase had broad substrate specificities in accepting picolinic acid and its analogues [4]. Cultures of ΔsanL strain were fed with 1 mM analogues of picolinic acid (benzoic acid, 4-hydroxybenzoic acid, nicotinic acid and isonicotinic acid). Cultures supplemented with benzoic acid produced nikkomycin Bx and Bz, which are consistent with the results from *S. tendae nikC* mutant [15]. When the cultures were supplemented with nicotinic acid, culture filtrates of ΔsanL strain regained the ability to inhibit the growth of *C. albicans* and *A. longipes* like the wild-type and complementary strains (Figure 2B and C). HPLC analysis revealed two distinct peaks at retention time 18.5 and 19.5 min (Figure 2A). In contrast, no obvious peak was detected at corresponding retention time in cultures supplemented with 4-hydroxybenzoic acid and isonicotinic acid except for a minor peak at retention time 22.2 min (Figure 2A). Bioassay was detected against *C. albicans* and *A. longipes*, no inhibition zone was observed (Figure 2B and C).

### Isolation and bioactive assay of nikkomycin Px and Pz

**Figure 3:**
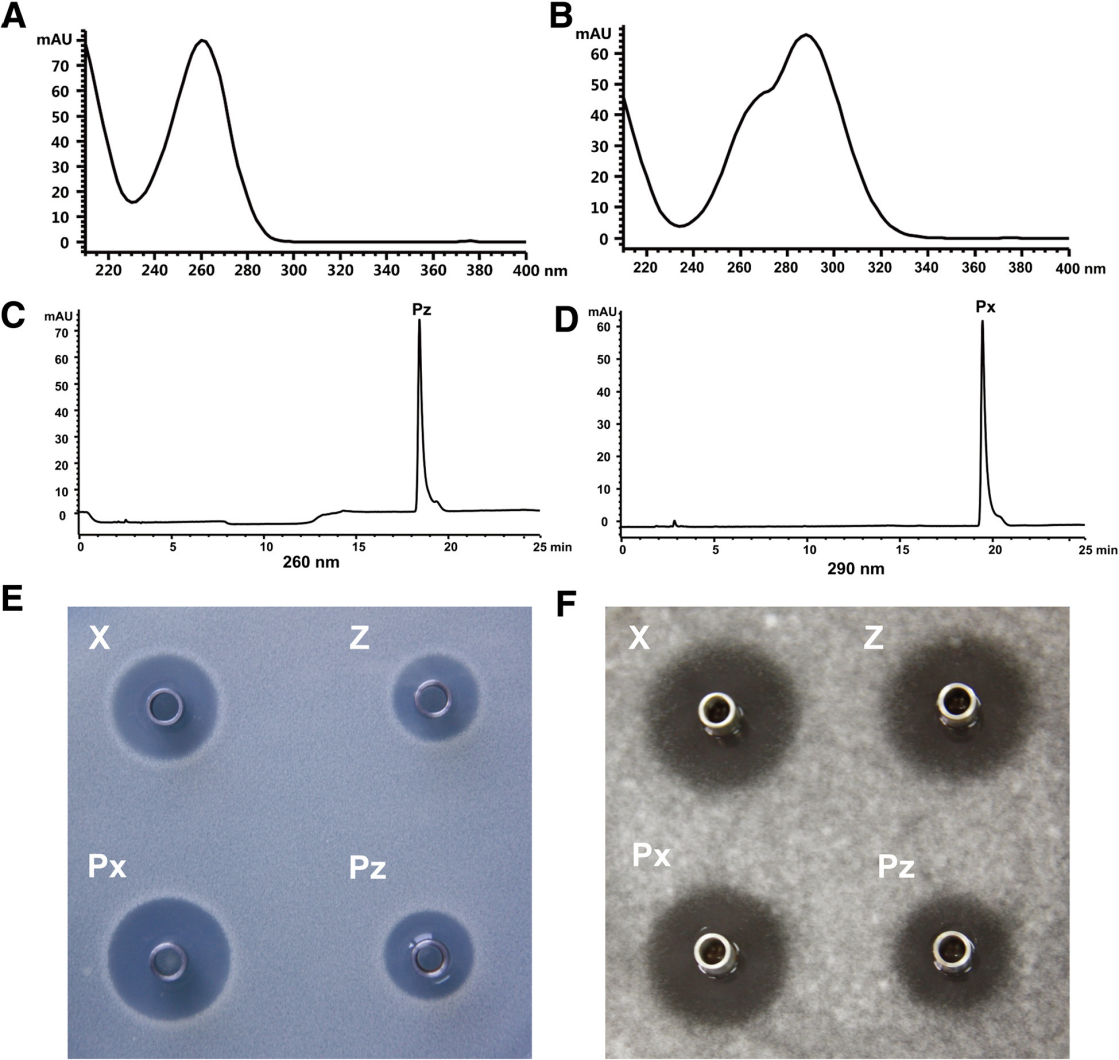
**Identification of nikkomycin Px and Pz.** UV spectrum of nikkomycin Pz and Px with an absorption maximum at 260 nm **(A)** and 290 nm **(B)**. HPLC analysis of purified nikkomycin Px **(C)** and Pz **(D)**. Bioassays of nikkomycin Pz and Pz against *C. albicans ***(E)** and *A. longipes ***(F)**. X: nikkomycin X, Z: nikkomycin Z, Px:nikkomycin Px, Pz: nikkomycin Pz.

In order to characterize the compounds responsible for antifungal activity, we isolated and purified them from 3 L broth of ΔsanL strain in fermentation tank. The two purified compounds (nikkomycin Px and Pz) are white powders, soluble in water but insoluble in ethanol, ether, ethyl acetate, methanol and acetone. When the compounds were dissolved in 0.1% trifluoroacetic acid (TFA) solution, nikkomycin Px and Pz showed maximum ultraviolet absorption at 290 nm and 260 nm, respectively (Figure 3A, B, C and D). In addition, the yields of two compounds were determined. It showed that ΔsanL strain supplemented with nicotinic acid can produce nikkomycin Px and Pz at 84 mg/L and 122 mg/L, respectively. The yield of nikkomycin Pz is close to that of nikkomycin Z (120 mg/L) in the wild-type strain, whereas the yield of nikkomycin Px is lower than that of nikkomycin X (220 mg/L) in the wild-type strain [16]. To compare their antifungal activities, five micrograms of each compound (nikkomycin X, Z, Px and Pz) were tested against *C. albicans* and *A. longipes*. Both nikkomycin Px and Pz exhibited comparatively strong inhibitory activity as nikkomycin X and Z produced by the wild-type strain (Figure 3E and F).

### Structural determination of nikkomycin Px and Pz

Structure modeling analysis indicated that nikkomycin Px and Pz may be new members of nikkomycin family incorporated with the supplemented substrate precursor, nicotinic acid, in the molecules. This assumption was further validated by electrospray ionization mass spectrometry (ESI-MS) and nuclear magnetic resonance spectroscopic (NMR) analysis.

**Figure 4:**
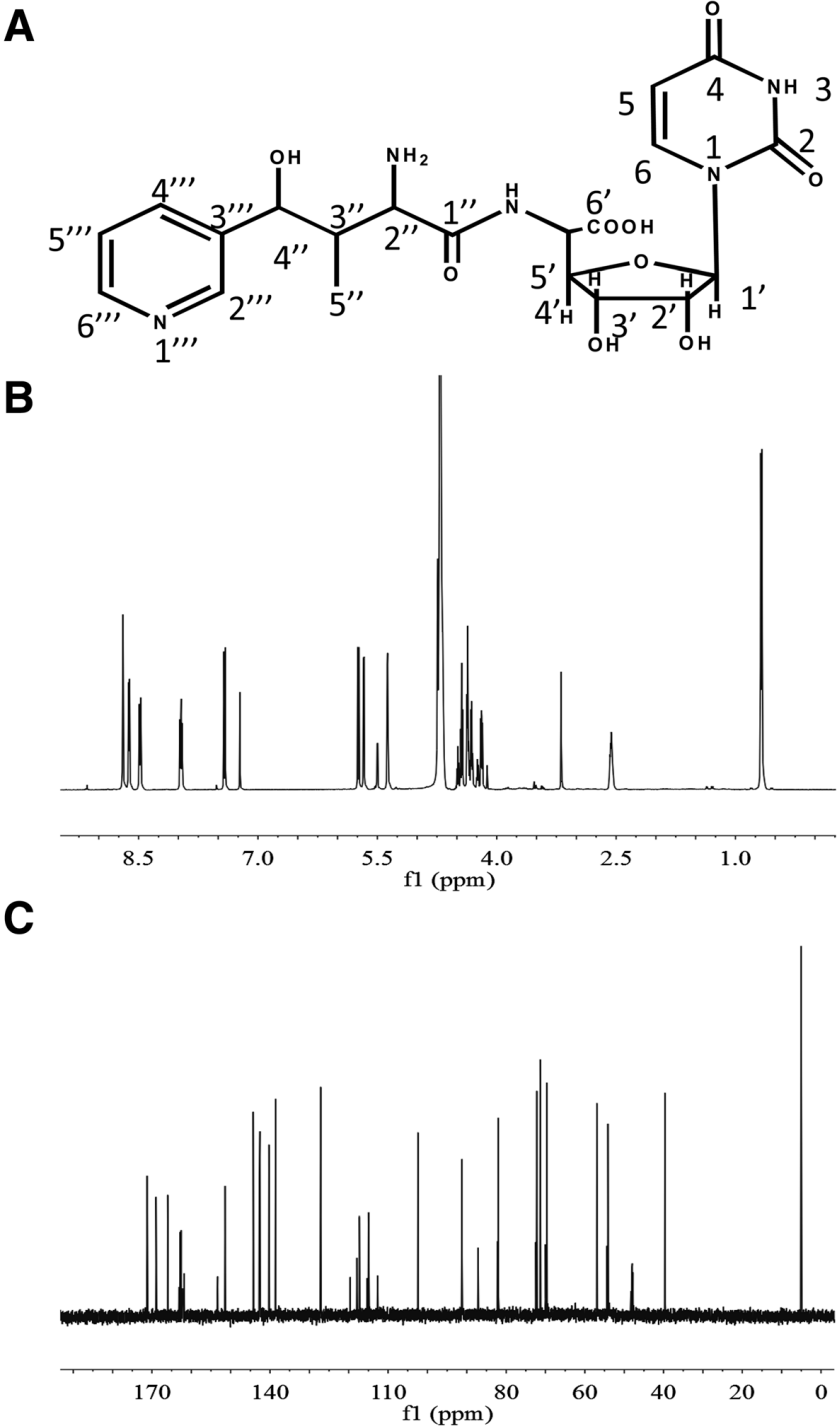
**Structural determination of nikkomycin Pz. (A)** Structure of nikkomycin Pz. **(B) **^1^H-NMR spectrum of nikkomycin Pz. **(C) **^13^C-NMR spectrum of nikkomycin Pz.

For nikkomycin Pz (Figure 4A), ESI-MS revealed an [M + H]^+^ ion at m/z 480.2 and a mass of 16 lower than nikkomycin Z, which are consistent with nikkomycin Z lacking a hydroxyl group from the pyridine ring. Molecular formula was subsequently established to be C_20_H_25_N_5_O_9_ (exact mass 479.2 Da) according to tandem mass spectrometry (MS/MS) and NMR data. By comparing ^1^H-NMR (Figure 4B) and ^13^C-NMR (Figure 4C) data to those of nikkomycin Z, ^1^H and ^13^C chemical shifts in nucleoside and homothreonine moieties were assigned (Table 1). A set of distinctive proton signals: δ8.68 (1H, s), δ8.46 (1H, d, *J* = 8.2Hz), δ7.94 (dd, *J* = 8.1Hz, 5.9Hz) and δ8.60 (1H, d, 5.7Hz) suggested the difference in pyridine ring. According to coupling constants (*J*) (Table 1), ^1^H-^1^H COSY (Additional file 1: Figure S1A) and ^1^H-^13^C HSQC data (Additional file 1: Figure S1B), it was concluded that the pyridinyl ring attached to C-4 of homothreonine (equivalent to C-4′′ in nikkomycin Pz) via *meta*-carbon. To differentiate C-4′′′ and C-6′′′ chemical shift, ^1^H-^13^C HMBC (Additional file 1: Figure S1C) was investigated. The cross-peak between ^13^C signal at δ144.3 and H-4” (δ5.36) suggested that the chemical shift of C-4′′′ and C-6′′′ is at δ144.3 and δ140.3 respectively. In summary, the nucleoside moiety in nikkomycin Pz is identical with that of nikkomycin Z, but the peptidyl moiety was determined as 4-(3′-pyridinyl)-homothreonine (equivalent to 4′′-(3′′′-pyridinyl)-homothreonine in nikkomycin Pz). The chemical shifts of nikkomycin Pz were summarized in Table 1.

**Table 1:** **Summary of **^**1**^**H and **^**13**^**C NMR data for nikkomycin Px and Pz in D**_**2**_**O**

**Position**	**Nikkomycin Px**		**Nikkomycin Pz**	
	**δ (**^**1**^**H, mult., *J*)**	^**13**^**C (δ)**	**δ (**^**1**^**H, mult., *J*)**	^**13**^**C (δ)**
2		153.4		151.4
4		124.1		166.0
5	7.53 (1H, s)	125.5	5.74 (1H, d, 8.1)	102.4
6	9.16 (1H, s)	180.3	7.42 (1H, d, 8.1)	142.5
1´	5.53 (1H, d, 4.5)	87.7	5.65 (1H, d, 4.2)	91.2
2´	4.43 (1H, dd, 4.5, 5.5)	72.3	4.29 (1H, dd, 4, 6)	72.2
3´	4.52 (1H, t, 5.5)	70.1	4.42 (1H, t, 6)	69.8
4´	4.25 (1H, dd, 4.0, 5.5)	82.3	4.17 (1H, dd, 3.5, 6.5)	82.0
5´	4.74 (1H, d, 4.0)	54.3	4.73 (1H, d, 4)	54.3
6´		171.2		171.3
1´´		168.9		168.9
2´´	4.36 (1H, d, 4)	57.0	4.35 (1H, d, 4)	56.9
3´´	2.36 (1H, m)	40.2	2.55 (1H, m)	39.6
4´´	5.37 (1H, s)	71.3	5.36 (1H, s)	71.3
5´´	0.68 (3H, d, 7)	5.11	0.67 (1H, d, 7.1)	5.05
2´´´	8.7 (1H, s)	138.5	8.68 (1H, s)	138.6
3´´´		142.8		142.7
4´´´	8.49 (1H, d, 8.2)	144.4	8.46 (1H, d, 8.2)	144.3
5´´´	7.97 (1H, dd, 8.1, 6)	127.1	7.94 (1H, dd, 8.1, 5.9)	127.1
6´´´	8.62 (1H, d, 5.5)	140.3	8.60 (1H, d, 5.7)	140.3

**Figure 5:**
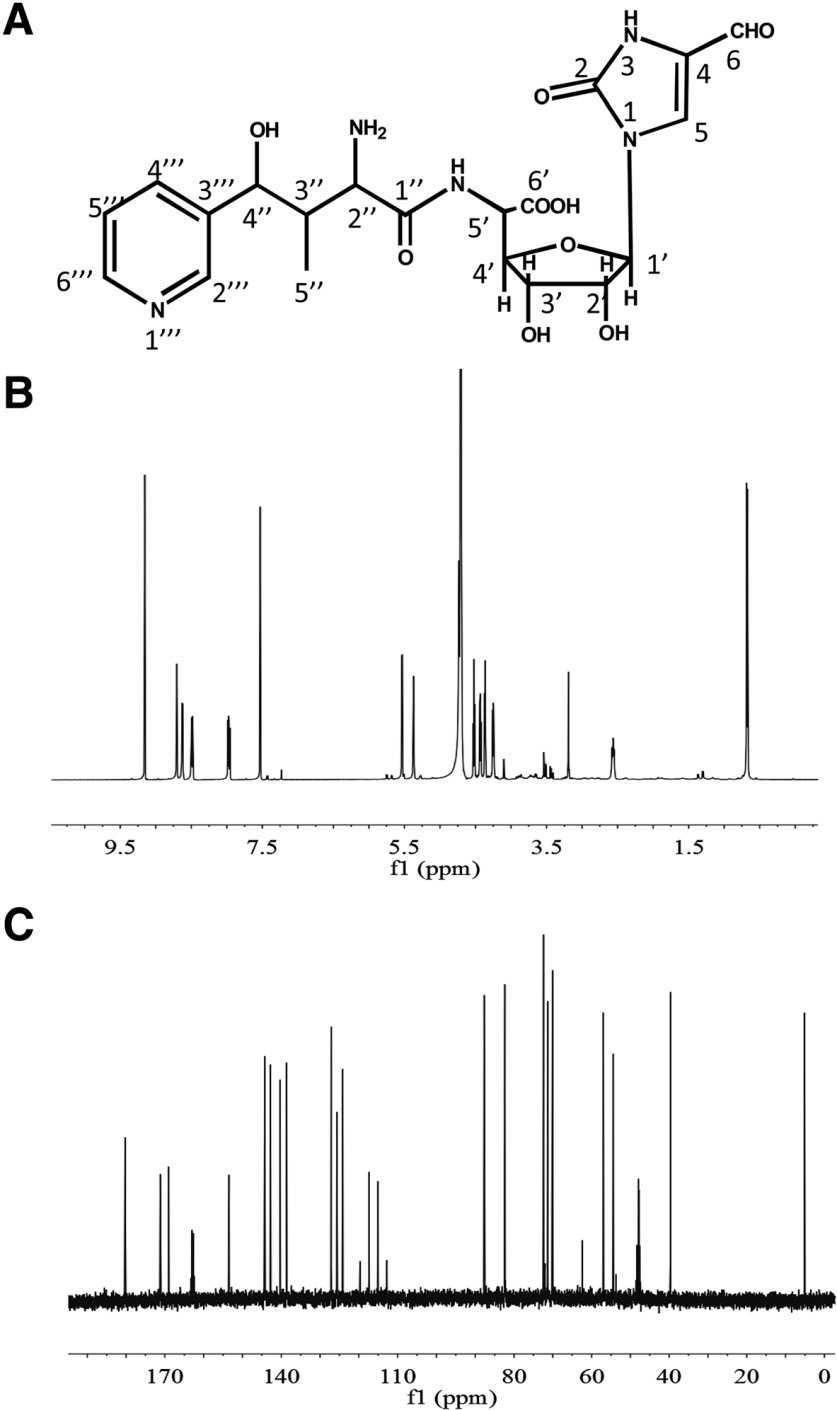
**Structural determination of nikkomycin Px. (A)** Structure of nikkomycin Px. **(B) **^1^H-NMR spectrum of nikkomycin Px. **(C) **^13^C-NMR spectrum of nikkomycin Px.

For nikkomycin Px (Figure 5A), ESI-MS analysis revealed the same fragmentation pattern as nikkomycin Pz, indicating the structure similarity between these two compounds. The molecular formula was determined as C_20_H_25_N_5_O_9_ (exact mass 479.2 Da) from MS/MS and NMR data. The identity of peptidyl and aminohexuronic acid moieties in both nikkomycin Px and Pz was confirmed by ^1^H-NMR (Figure 5B), ^13^C-NMR (Figure 5C) and ^1^H-^1^H COSY (Additional file 1: Figure S2A) data. The comparison of NMR to those of nikkomycin X indicated that the 4-formyl-4-imidazoline-2-one could be the base of nucleoside moiety in nikkomycin Px. H-5 signal at δ 5.74 in nikkomycin Pz disappeared in nikkomycin Px and consequently the doublet of H-6 (δ7.39, d, 8.1) in Pz has changed to singlet (δ7.53, s) in nikkomycin Px. A clear proton signal at δ9.16 (1H, s) and carbon signal at δ180.3 in nikkomycin Px suggested an aldehyde group. ^1^H and ^13^C signals of the base group were finally assigned by ^1^H-^13^C HSQC data (Additional file 1: Figure S2B) and ^1^H-^13^C HMBC data (Additional file 1: Figure S2C). The nucleoside moiety in nikkomycin Px was corroborated as aminohexuronic acid with N-glycosidically bonded 4-formyl-4-imidazoline-2-one in nikkomycin Px. Thus, it was confirmed that nikkomycin Px shares the same nucleoside moiety with nikkomycin X, but its peptidyl moiety was determined as 4-(3′-pyridinyl)-homothreonine (equivalent to 4′′-(3′′′-pyridinyl)-homothreonine in nikkomycin Px). Chemical shifts of nikkomycin Px were summarized in Table 1.

### Stability of nikkomycin Px and Pz under different pHs and temperatures

**Figure 6:**
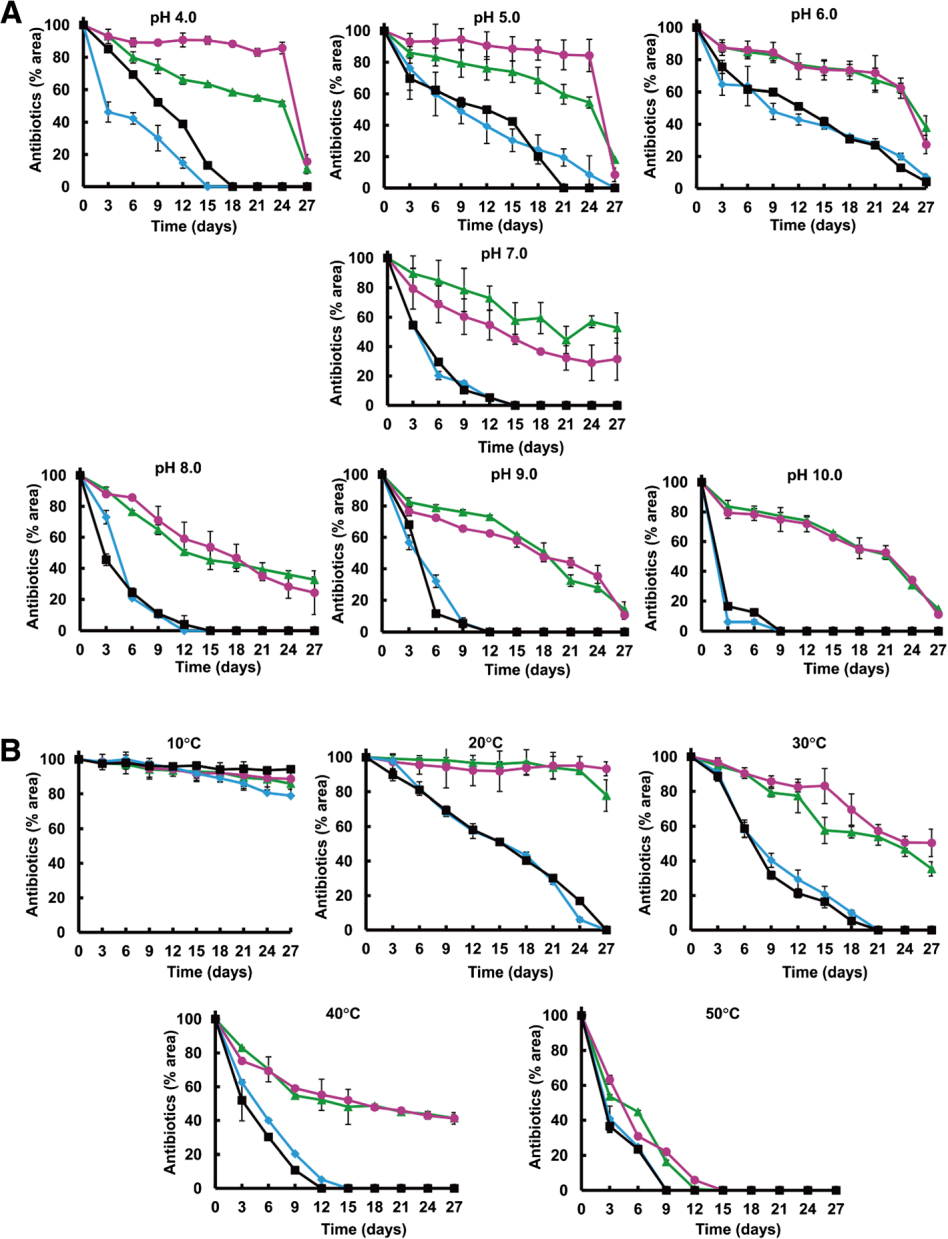
**Stability of nikkomycin Px(Pink circle), Pz(Green triangle), X(Black square) and Z(Blue diamond) under different pHs (A) and temperatures (B).** All samples were analyzed by HPLC and quantified according to the areas of peaks.

The stability of nikkomycin Px and Pz was performed. They were more stable than nikkomycin X and Z at pH from 4 to 10. Under acid conditions (pH4, 5 and 6), 60-80% nikkomycin Px and Pz were present after 24 days of incubation whereas only 0-20% nikkomycin X and Z were remained (Figure 6A). Under neutral and alkaline conditions (pH7, 8, 9 and 10), 40% nikkomycin Px and Pz were present after 24 days of incubation while nikkomycin X and Z were completely degraded (Figure 6A).

No obvious instability of the four compounds was observed at 10°C, but all of them became less stable with increasing temperatures (Figure 6B). The stability of nikkomycin Px and Pz is much better than that of nikkkomycin X and Z.

## Discussion

Owing to their low toxicity to mammals and bees, nikkomycins are considered as competitive inhibitors of chitin synthetases in fungi, insects, acarids and yeasts [3]. During the past several decades, extensive studies have been carried out to elucidate the biosynthetic pathway of nikkomycins [4,7-11]. Based on prior knowledge, novel nikkomycin analogues can be produced by generating mutant deficient in formation of biosynthetic intermediates and subsequently feeding the mutant with a variety of alternative intermediates.

Two novel nikkomycin analogues (nikkomycin Px and Pz) were generated by supplementation of *S. ansochromogenes* ΔsanL with nicotinic acid. Unlike nikkomycin X and Z, nikkomycin Px and Pz contain 4-(3′-pyridinyl)-homothreonine as the peptidyl moiety. As expected, the position of nitrogen in the pyridinyl ring was changed due to the difference between nicotinic acid and picolinic acid. Surprisingly, the hydroxyl group is absent in the pyridinyl ring of nikkomycin Px and Pz (Figures 4A and 5A). Previous study in our lab demonstrated that SanH and SanI are responsible for the hydroxylation of pyridinyl residue in nikkomycin X and Z [17]. The lack of the hydroxyl group in the pyridinyl ring of nikkomycin Px and Pz indicated that SanH and SanI are unable to recognize substrate with 3′-pyridinyl ring.

Antifungal activities of nikkomycin Px and Pz are similar to those of nikkomycin X and Z (Figure 2B and C). This result indicated that incorporation of nicotinic acid into the peptidyl moiety of nikkomycins has no effect on their biological activities. However, nikkomycin Px and Pz displayed better stabilities than nikkomycin X and Z under different pHs and temperatures (Figure 6). This was attributed to the combined effect of change of nitrogen position and loss of hydroxyl group in the pyridinyl ring. Our results reinforced the proposal that the stability of nikkomycins was influenced by the peptidyl moiety of the compounds [15,18].

## Conclusions

Two novel nikkomycin analogues (nikkomycin Px and Pz) were generated by mutasynthesis with *S. ansochromogenes* ΔsanL strain. Nikkomycin Px and Pz showed comparable antifungal activity as nikkomycin X and Z. Moreover, they also displayed better stabilities than nikkomycin X and Z under different pHs and temperatures.

## Materials and methods

### Strains and culture conditions

*Streptomyces ansochromogenes* 7100 deposited at China General Microbiological Culture Collection (CGMCC) is a natural nikkomycin producer. *Candida albicans* and *Alternaria longipes* from CGMCC were used as indicator strains for nikkomycin activity bioassay [11]. *E. coli* DH5α was used for propagating plasmids. *E. coli* ET12567 (pUZ8002) was used for conjugal transfer of DNA from *E. coli* to *Streptomyces*[19].

*Streptomyces* strains were grown on R2YE medium, mannitol soya flour (MS), minimal medium (MM) or in yeast extract-malt extract (YEME) liquid medium at 28°C. The culture conditions for nikkomycin production were essentially as described previously [16]. In brief, spore suspensions were inoculated in liquid YEME and incubated at 28°C on a rotary shaker (220 rpm) for 48 hr as seed culture. A total of 1 ml seed culture was transferred into 100 ml SP medium (3% mannitol, 1% soluble starch, 0.75% yeast extract, and 0.5% soy peptone, pH 6.0) [20] for nikkomycin production. When necessary, apramycin or kanamycin was added at concentration of 50 μg/ml for R2YE, 7 μg/ml for MM and YEME.

### Construction of ΔsanL and its complementation

For the construction of ΔsanL in *S. ansochromogenes*, a 2.1 kb *Bgl*II-*Bst*XI DNA fragment containing complete *sanL* was blunted and inserted into the *Eco*RV site of pBluescript II KS(+). The resulting plasmid was linearized with *Bcl*I and blunted with Mung bean nuclease. The kanamycin-resistance gene (*neo*) was obtained from pUC119::*neo* after digestion with *Bam*HI and *Kpn*I, blunted and ligated into the blunted *Bcl*I site of *sanL* to generate pBS-L::*neo*. A 3.1 kb insert of pBS-L::*neo* was isolated after digestion with *Hin*dIII and *Eco*RI, and the recovered fragment was then ligated into the same sites of pKC1139. The resulting pKC1139L::*neo* was confirmed by restriction digestion and then introduced into *S. ansochromogenes* 7100 by intergeneric conjugation from *E. coli* ET12567 (pUZ8002) according to standard techniques [21]. The resulting transformants were inoculated on agar MM to form spores. Gray spores were harvested and spread on agar MM containing kanamycin as resistance selection. After incubating at 40°C for 3 days, the *sanL* disruption mutants were selected with colonies exhibiting kanamycin resistance (Kan^r^) and apramycin sensitivity (Apr^s^). Kan^r^/Apr^s^ strains were further verified by PCR and Southern blot analysis. The confirmed *sanL* disruption mutant was designated as ΔsanL.

For complementation experiments, the constitutive *hrdB* promoter was used to drive the expression of *sanL*. The *hrdB* promoter was amplified from genomic DNA of *Streptomyces coelicolor* M145 with primers hrdB-pF/hrdB-pR (5′-aattagatctCCGCCTTCCGCCGGAACG-3′; 5′-GAACAACCTCTCGGAACGTTGA-3′). The coding region of *sanL* was amplified from genomic DNA of *S. ansochromogenes* 7100 with primer pair sanL-cF/sanL-cR (5′-ATGCTGACCGTGAACGGGAACTC-3′; 5′-attgaattcTCATGCCCGGGCCTCCTCG-3′). Prior to PCR amplification, hrdB-pR was phosphorylated with T4 polynucleotide kinase to facilitate subsequent ligation reactions. The *hrdB* promoter fragment was digested with *Bgl*II, and the *sanL*-coding region was digested with *Eco*RI. Both the *hrdB* promoter and *sanL*-coding fragment were ligated together with *Bam*HI/*Eco*RI double-digested pSET152. The resulting pSET152::*sanL* was introduced into the ΔsanL strain to generate the complementation strain sanLc.

### Detection of nikkomycins and antifungal bioassays

For the analysis of nikkomycin, culture broths were centrifuged and the supernatants were filtered through a millipore membrane (pore diameter 0.22 μm). HPLC analysis was performed with Agilent 1100 HPLC system and ZORBAX SB C-18 (5 μm, 4.6 × 250 mm). Chemical reagent, mobile phase and gradient elution process were as described previously [22]. The elution was detected with photodiode array at 260 and 290 nm for nikkomycins. Bioassays against *C. albicans* and *A. longipes* were carried out by a disk diffusion method as described previously [5]. The procedure is essentially the same for both indicator strains except that an overnight culture of *C. albicans* was used while 5 day-old culture was used for *A. longipes*. The modified potato dextrose agar medium (20% potatoes, 2% glucose and 0.8% agar) was heated to dissolve and then cooled to 50°C before use. Cultures of indicator strains (50 μl for *C. albicans* and 10 ml for *A. longipes*) were well dispersed in 100 ml pre-dissolved medium and poured to a 15 cm plate. Oxford cups were placed onto the plates and antibiotics were then added into the Oxford cups. After incubation for 12–24 h at 28°C, the zone of inhibition was assessed.

### Isolation of nikkomycin Px and Pz

The method for isolation of nikkomycin Px and Pz was similar to that for polyoxin P [23]. Briefly, seed culture of *S. ansochromogenes* ΔsanL was prepared as mention above. A total of 30 ml seed culture transferred into 3 L SP supplemented with 1 mM nicotinic acid. After 5 days of fermentation, the culture broth of *S. ansochromogenes* ΔsanL was harvested by centrifugation and the supernatant was adjusted to pH 4.5 with acetic acid. The sample was then chromatographed on a macroporous absorption resin HP-20 (Mitsubishi) column, and the flow-through was collected and subjected to a Dowex 50WX2 (Sigma) column. The column was eluted with 0.4 N ammonia solution and fractions with antifungal activity was collected and concentrated to a small volume *in vacuo*. After addition of 6 volumes of cold ethanol, the precipitate was collected by centrifugation. The dried powder was subsequently dissolved in water and further purified by HPLC.

### Stability determination of nikkomycin X, Z, Px and Pz

To dissect their stabilities under different pH conditions, the four antibiotics (nikkomycin X, Z, Px and Pz) were dissolved in buffers with different pH values: 0.2 M CH_3_COONa buffer (pH adjusted to 4.0 or 5.0 with acetic acid), 0.05 M KH_2_PO_4_ buffer (pH adjusted to 6.0 and 7.0 with NaOH), 0.05 M Tris buffer (pH adjusted to 8.0 with HCl), 0.025 M Na_2_CO_3_ buffer (pH adjusted to 9.0 and 10.0 with NaOH). All samples were incubated at 25°C for 27 days. To dissect the effect of temperature on their stabilities, antibiotics were dissolved in 0.05 M KH_2_PO_4_ buffer (pH 6.0) and incubated at 10, 20, 30, 40 and 50°C for 27 days. Residual antibiotics were quantified by the peak areas.

### Spectrometric analyses

MS analysis and tandem mass spectrometry analysis were carried out on Triple-Quadrupole LC-MS/MS (Agilent 1260/6460) in positive mode. All NMR spectra were recorded on a Bruker Advance spectrometer (AV500 MHz).

## Competing interests

The authors declare that they have no competing interests.

## Authors’ contributions

CF carried out experiments and analyzed the primary data. HL constructed *sanL* mutant and assisted with feeding experiments. DD assisted with data analysis of MS and NMR. JZ assisted with data analysis of MS, NMR and revised the manuscript. GN wrote and revised the manuscript. HT supervised the whole research work and revised the manuscript. All authors read and approved the final manuscript.

## Supplementary Material

Additional file 1: Figure S1 NMR Spectra of nikkomycin Pz. (A) ^1^H-^1^H COSY spectrum of nikkomycin Pz. (B) ^1^H-^13^C HSQC spectrum of nikkomycin Pz. (C) ^1^H-^13^C HMBC spectrum of nikkomycin Pz. **Figure S2.** NMR Spectra of nikkomycin Px. (A) ^1^H-^1^H COSY spectrum of nikkomycin Px. (B) ^1^H-^13^C HSQC spectrum of nikkomycin Px. (C) ^1^H-^13^C HMBC spectrum of nikkomycin Px. Click here for file
