# Supplementary material for: Novel nikkomycin analogues generated by mutasynthesis in Streptomyces ansochromogenes
Source: Microb Cell Fact. 2014 Apr 21;13:59. doi: 10.1186/1475-2859-13-59 (PMC4021061; doi:10.1186/1475-2859-13-59)

Figure S1 NMR Spectra of nikkomycin Pz. (A) ^{1}H-^{1}H COSY spectrum of nikkomycin Pz. (B) ^{1}H-^{13}C HSQC spectrum of nikkomycin Pz. (C) ^{1}H-^{13}C HMBC spectrum of nikkomycin Pz.

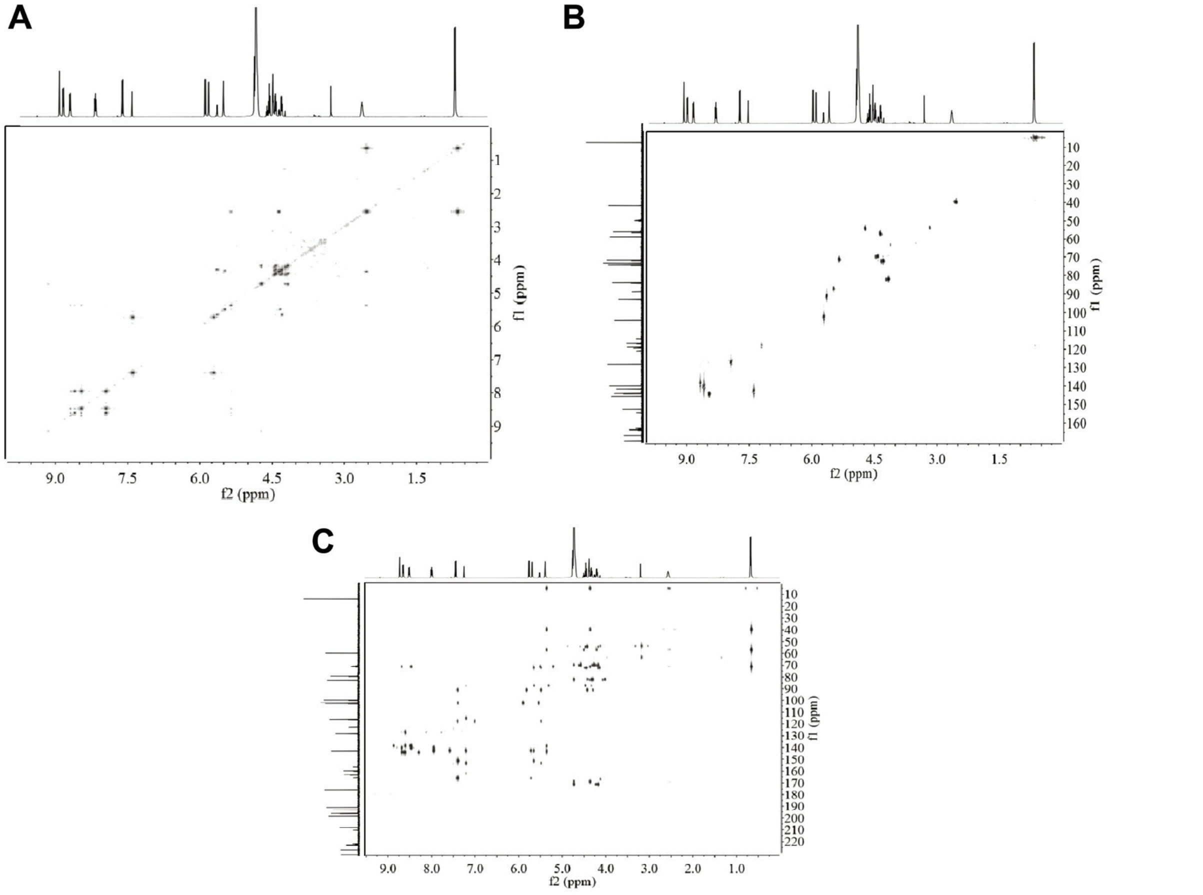

Figure S2 NMR Spectra of nikkomycin Px. (A) ^{1}H-^{1}H COSY spectrum of nikkomycin Px. (B) ^{1}H-^{13}C HSQC spectrum of nikkomycin Px. (C) ^{1}H-^{13}C HMBC spectrum of nikkomycin Px.

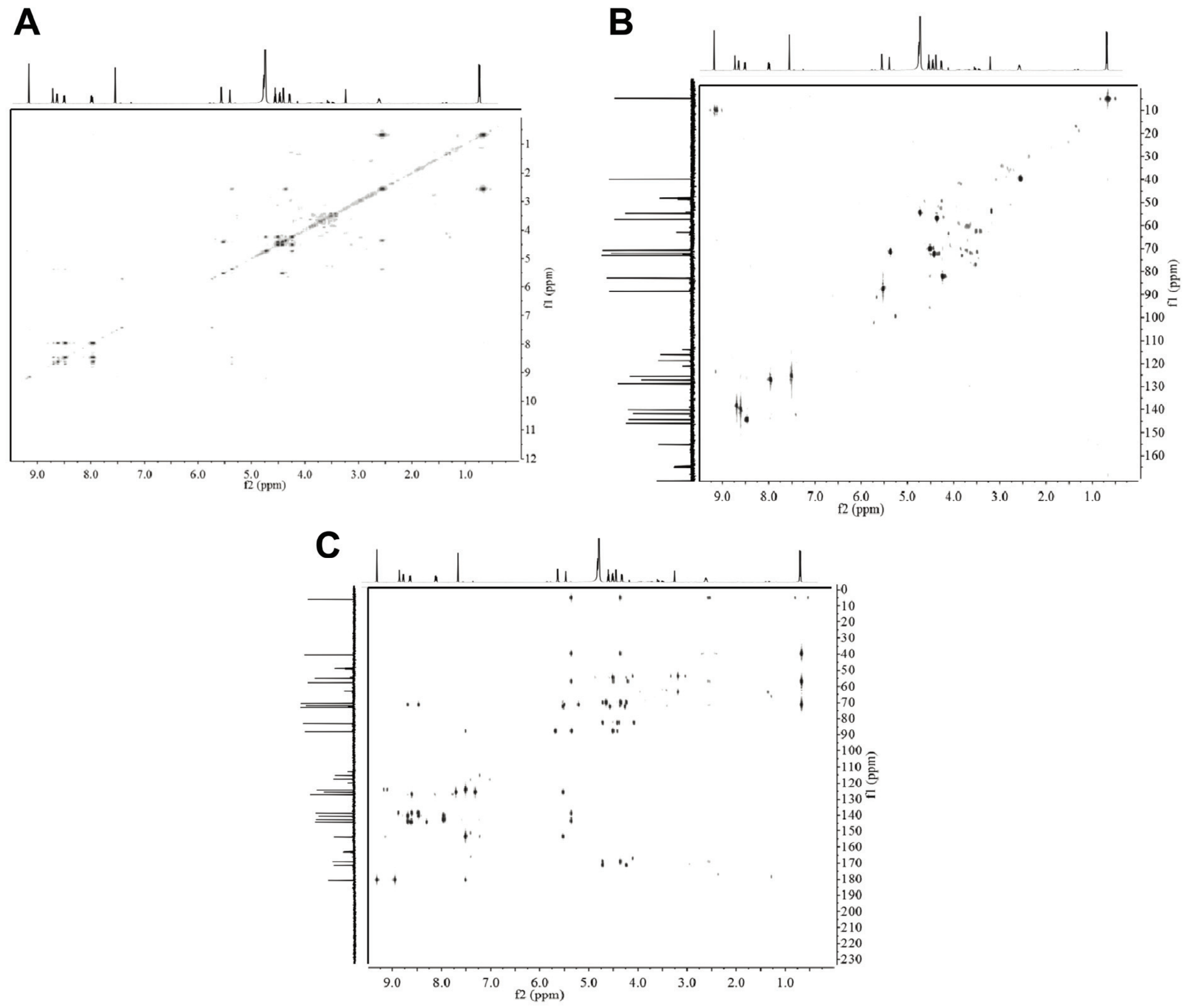

Supplement: Additional file 1: Figure S1 — NMR Spectra of nikkomycin Pz. (A) 1H-1H COSY spectrum of nikkomycin Pz. (B) 1H-13C HSQC spectrum of nikkomycin Pz. (C) 1H-13C HMBC spectrum of nikkomycin Pz. Figure S2. NMR Spectra of nikkomycin Px. (A) 1H-1H COSY spectrum of nikkomycin Px. (B) 1H-13C HSQC spectrum of nikkomycin Px. (C) 1H-13C HMBC spectrum of nikkomycin Px. [file 1475-2859-13-59-S1.pdf]
